# Progress in Surgery

**Published:** 1900-01-20

**Authors:** 


### PROGRESS IN SURGERY.

### TUMOURS.

**Colloid Cancer.**—Drs. Kelly and Emery[1] have reported an interesting case of this affection. The chief points in the history were as follows: Mr. B. Hill, seven and a half years before her death, removed the greater part of the right breast for a growth which was considered to be a duct-papilloma. An illustration in Erichsen's "Surgery," tenth edition, was obtained from it. There was recurrence in four and a half years, but this was not operated upon until six years after the first operation. There were no enlarged glands at this time. Sections of the recurrent growth showed it to be composed chiefly of colloid material, with small clusters of degenerated cells. At one part spheroidal cells surrounded by fibrous tissue existed. Twelve months later a second recurrent growth was removed, and found to be a typical colloid carcinoma. No other growth was discovered at this time, but the patient had pain in the right hip and leg, thought to be rheumatic. She rapidly lost ground after this, and died in five months. During the latter months there was marked cachexia, pain in the right hypochondrium and in the leg, enlargement of the liver, troublesome cough, and a very curious condition developed in the right half of the frontal bone, with intense headache. A depression formed in that bone two inches in diameter, and nearly circular. The edges were irregular, but not at all raised, and through the depression the pulsations of the brain could be felt, the bone being replaced by a thin tense membrane. Pressure on this area was intensely painful. The depth of the depression was one and a half inches. The skin over it was normal and non-adherent. A secondary growth formed in the sternum also. At the autopsy in the lung were found scattered colloid nodules, in the liver nodules like those of scirrhus, and in the manubrium a tumour like soft cartilage. The membrane which had replaced a portion of the frontal bone was one-twentieth of an inch thick, and adherent to the dura mater, and the edges of bone around it were infiltrated with new growth, but without being thickened. The colloidal degeneration reached its maximum in the sternum, and there was no trace of it in any part of the skull bones examined nor in the membrane. There was rarifaction of the edges of the skull bones, with cancerous alveoli in the tissue filling the spaces. In the membrane were similar alveoli, but filled only with nuclei of degenerate type, as though at this part the new growth first replaced the bone and then degenerated into a membrane.

Dr. Ferguson[2] reports two cases of colloid cancer in the abdomen. In the first, a man of 60, laparotomy revealed a large quantity of colloid material in the peritoneal cavity, some of it free, some contained in slender vesicles. The peritoneum was studded with these. The second case was that of a woman of 55, who was found to have also a large quantity of colloid material in the enlarged abdomen and a firm cancerous mass in the right iliac fossa, and other small firm masses of growth scattered over the peritoneum. He remarked that the epithelioid cells in the nests invaded by colloid change were not all changed. Hence when rupture occurred, and the glue-like material escaped, it carried with it cells still retaining their original anatomical structure, which was interesting from the point of view of the dissemination. He remarked also that, in spite of the great frequency of cancer of the female generative tract, only 28 cases of cancer of the penis were recorded in men whose wives were so affected.

**Cases of Special Interest.**—J. B. Davey[3] reports a case of acute carcinoma of the breast. The patient, a woman of 29, noticed nothing wrong with her left breast until her baby, seven months old, refused to take the nipple. At this time the patient could discover nothing wrong with the breast. Two months later the nipple was retracted and the whole breast converted into a firm mass, and around it were numerous small nodules in the skin, and there were enlarged glands in the axilla. After another month she died. At the post-mortem examination the other breast was found to be extensively infiltrated with new growth, also the body of the sternum. Numerous cancer nodules were found in the pleuræ, liver, supra-renals, and the lumbar vertebræ. Any case of carcinoma of the breast terminating under a year is exceptional, but the duration in this case was only three months. Lactation always increases the rate of growth. Mr. Gould has found that the influence of pregnancy on the course of the disease is the reverse of that of lactation, the disease often making slower progress in pregnancy, and, after delivery, advancing with extreme rapidity. It is unusual for the supra-renals to be involved. The child had not refused the right breast even when involved, and no doubt had taken the left breast until the disease became far advanced.

Dr. Stanley[4] relates a case of primary carcinoma of the liver. The patient was a man of 29. Three months before death he became liable to attacks of "indigestion," with vomiting, and epigastric pain. Six weeks after the beginning of these symptoms he noticed a lump in the abdomen on the right side. He was admitted to hospital 13 days before death, primarily for varicocele, and then the existence of an enlarged liver was discovered. The lower border of the liver reached the umbilicus. He died rather suddenly. The liver was found studded with yellowish grey nodules of various sizes. The gall-bladder was free. There were a few secondary nodules in each lung, but the whole alimentary tract and all other organs were free. The large nodules microscopically showed irregular columns of epithelial-like cells in a fibrous stroma.

G. Reynaud[5] reports a case of general cancerous infection from the stomach, in a man of 39. For four months he had suffered from a slow and painful digestion, nausea, occasional vomiting, and constant constipation. Later symptoms of pleurisy, of ascites, of anasarca, and of cachexia supervened. He died after an illness of nine months at the most. Starting at the pylorus, the cancerous process, colloid in nature, had invaded the whole stomach, the great omentum, mesentery, intestinal coils, head of pancreas, spleen, kidneys, vertebræ, liver, lungs, and pleuræ. The lymphatic route was probably that of the dissemination; possibly the ascitic fluid had served as a vehicle also.

**Carcinoma of the Parotid Gland.**—Dr. C. K. Briddon[6] reports the case of a woman of 25 years of age, who about seven years ago first noticed a small lump over the left parotid gland. It gave no inconvenience, but gradually enlarged for four years, reaching the size of a lemon. It was then excised, the pathological report being—mixed sarcoma and carcinoma. The growth reappeared in a year, and was again removed. In six months it appeared again, and grew much more rapidly, and was again removed at the end of 15 months. She had then constant aching pain in it. Its size was two and a half by three inches in diameter, and it was partly in front of and partly behind the ear, which was elevated by it. A complete extirpation of the diseased parotid gland was performed, together with the external auditory canal, but leaving the auricle. The skin over it was removed. Healing was assisted by Thiersch's skin grafts. The microscope showed it to be a carcinoma. The cells were quite small, the alveoli in places very long and narrow.

**Chondro-Carcinoma of the Testicle.**—A. Caddy publishes an account of a case of this kind[6] from India. The patient was a European. In December, 1894, he was struck with a cricket ball on the right testicle. A week later swelling and hardness in the lower part was first noticed. This did not alter until 10 months later, when the hardness began to spread upwards and the testicle to increase in size. Caddy first saw him 12 months later, and found the testicle the size of a duck's egg, painless, and hard. The enlargement then remained stationary for nearly two years, when pain came on for the first time, and suddenly the testicle began to enlarge again, and a fortnight later was removed. The cord was not thickened, and no iliac or groin glands were enlarged. On section of the testicle, which was the size of a closed fist, no testicular tissue was apparent to the naked eye, the whole gland appearing infiltrated with growth. At the upper and back part a nodule of cartilage was cut through the size of a nutmeg. Major Evans, Professor of Pathology at the Calcutta Medical College, reported that the testicle was the seat of a chondro-carcinoma. The cartilage was located in the neighbourhood of the epididymis, and was surrounded and interrupted by many cysts, lined by columnar epithalium. It is interesting that for nearly four years the disease remained quiescent, and then began to grow rapidly.

**Polyadenomata of the Large Intestine.**—Quénu and Landel[7] deal at considerable length with a form of adenoma of the large intestine, differing in many respects from the more frequent solitary polypus met with usually in young subjects and considered always to be of a benign character. In polyadenomata, known to surgeons here as multiple adenoma of the colon and rectum, and as disseminated polypi, the growths are abundant and scattered along the whole of the inner surface of the large intestine. They frequently coexist with intestinal cancer. In no fewer than 20 out of 42 cases collected by the authors the patients had also suffered from cylindrical epithelioma. The malignant complication was of later date than the adenomata, and probably due to cancerous degeneration of one or more of the polypoid growths. No period of life is exempt. Mostly, they occur between the ages of 16 and 30 years. The prognosis is grave, not only from the frequent association with cancer, but also from the abundant and persistent diarrhœa, the continuous loss of blood, and the severe abdominal pains.

[1] Lancet, May, 1899. [2] Med. Rec., June, 1899. [3] Treatment, May, 1899 [4] Birm. Med. Rev., Sept., 1899. [5] Brit. Med. Jour., July, 1899. [6] Annal of Surgery, Aug., 1899. [7] Brit. Med. Jour., June, 1899.

